# mTOR Inhibitor Rapalink-1 Prevents Ethanol-Induced Senescence in Endothelial Cells

**DOI:** 10.3390/cells12222609

**Published:** 2023-11-11

**Authors:** Huakang Zhou, Xuanchen Li, Majeed Rana, Jan Frederick Cornelius, Dilaware Khan, Sajjad Muhammad

**Affiliations:** 1 Department of Neurosurgery, Medical Faculty, University Hospital Düsseldorf, Heinrich-Heine-University, Moorenstrasse 5, 40225 Düsseldorf, Germanysajjad.muhammad@med.uni-duesseldorf.de (S.M.); 2 Department of Oral and Maxillofacial Surgery, Medical Faculty, Heinrich-Heine-University, Moorenstrasse 5, 40225 Düsseldorf, Germany; 3 Department of Neurosurgery, University Hospital Helsinki, Topeliuksenkatu 5, 00260 Helsinki, Finland; 4 Department of Neurosurgery, King Edward Medical University, Lahore 54000, Pakistan

**Keywords:** ethanol, endothelial cells, senescence, SASP, Rapalink-1, NFκ-B, MAPKs, mTOR

## Abstract

The cardiovascular risk factors, including smoking, ethanol, and oxidative stress, can induce cellular senescence. The senescent cells increase the expression and release of pro-inflammatory molecules and matrix metalloproteinase (MMPs). These pro-inflammatory molecules and MMPs promote the infiltration and accumulation of inflammatory cells in the vascular tissue, exacerbating vascular tissue inflammation. MMPs damage vascular tissue by degenerating the extracellular matrix. Consequently, these cellular and molecular events promote the initiation and progression of cardiovascular diseases. We used Rapalink-1, an mTOR inhibitor, to block ethanol-induced senescence. Rapalink-1 inhibited oxidative-stress-induced DNA damage and senescence in endothelial cells exposed to ethanol. It attenuated the relative protein expression of senescence marker P21 and improved the relative protein expression of DNA repair protein KU70 and aging marker Lamin B1. It inhibited the activation of NF-κB, MAPKs (P38 and ERK), and mTOR pathway proteins (mTOR, 4EBP-1, and S6). Moreover, Rapalink-1 suppressed ethanol-induced mRNA expression of ICAM-1, E-selectin, MCP-1, IL-8, MMP-2, and TIMP-2. Rapalink-1 also reduced the relative protein expression of MMP-2. In summary, Rapalink-1 prevented senescence, inhibited pro-inflammatory pathway activation, and ameliorated pro-inflammatory molecule expression and MMP-2.

## 1. Introduction

Endothelial cells form the inner monolayer of blood vessels, functioning as a critical interface between tissue and blood. Vascular endothelial cells can be suggested as the first cell type, which responds to hemodynamic stress and noxious stimuli such as acquired cardiovascular risk factors, including smoking, alcohol abuse, obesity, and increased lipid profile. In response to hemodynamic stress, smoking, alcohol abuse, oxidative stress, and increased lipid profile, endothelial cells are activated and gradually become dysfunctional and senescent [1,2,3]. Cellular senescence is characterized by four independent characteristics, i.e., cell cycle withdrawal, macromolecular damage, secretory phenotype, and deregulated metabolism [4,5]. Senescence is of two types: replicative senescence and stress-induced premature senescence [6]. Premature senescence can result from mitochondrial dysfunction, oncogene, and DNA damage induced by oxidative stress, radiation, alcohol, etc. [1,2,3,6,7]. There are contradictory reports on the impact of ethanol on senescence. On one hand, ethanol has been shown to induce senescence and reduce the differentiation potential of stem cells in vitro [8], while, on the other hand, it has been shown to be beneficial in low doses, reducing senescence in experimental animal studies [9].

The senescent cells remain metabolically active and acquire a pro-inflammatory state called senescence-associated secretory phenotype (SASP) [7]. These cells increase the expression and release of pro-inflammatory cytokines, chemokines, cell adhesion molecules, and matrix metalloproteinase (MMPs) [1,2,3]. SASP factors contribute to the pathogenesis and development of atherosclerosis [10]. The expression and release of SASP factors, including cell adhesion molecules and chemokines, promote tissue infiltration of inflammatory cells, resulting in an inflammatory microenvironment surrounding senescent cells [10,11]. In addition to that, senescent cells recruit their neighboring healthy cells towards senescence through a mechanism called paracrine senescence [7]. The senescent cells are eliminated by immune cells through a process called efferocytosis [6]. With increasing age, the capacity of immune cells to phagocytose senescent cells decreases, resulting in their accumulation in different tissues and organs, including vascular tissue [7]. This results in low-grade sterile chronic inflammation [7,12] that subsequently contributes to the initiation and progression of cardiovascular diseases. The accumulation of senescent cells has been observed in atherosclerotic lesions and intracranial aneurysm (IA) tissue [6,7,13], and by promoting plaque vulnerability, senescent cells can contribute to myocardial infarction and stroke [6]. Removal of senescent cells by applying different strategies has, so far, reduced the progression of IA and atherosclerotic lesions and decreased atherosclerotic plaque rupture [6,13].

Cellular senescence and SASP are regulated by NF-κB, MAPKs, and mTOR pathways [10,11,14]. Oxidative stress and ethanol treatment can activate these pathways [1,3,14]. The expression of SASP factors is regulated by MAPKs and mediated through NF-kB transcriptional activity [14]. Blocking NF-κB, MAPKs, and mTOR could inhibit cellular senescence and SASP and increase life span [15,16,17,18,19]. Moreover, activation of these pathways has been implicated in cardiovascular diseases, including atherosclerosis and IAs [15,20,21,22,23]. Animal experimental studies have shown that blocking the activation of these pathways could reduce the formation and progression of atherosclerosis and IAs [15,20,21,22,23].

In this study, we used Rapalink-1 to attenuate ethanol-induced endothelial cell senescence and SASP. Also, the impact of Rapalink-1 on the regulation of NF-κB, MAPKs, and mTOR pathways was investigated in endothelial cells exposed to ethanol.

## 2. Material and Methods

### 2.1. Cell Culture

HUVECs (Promocell, Heidelberg, Germany) were maintained in an endothelial cell medium (C-22010, Promocell, Heidelberg, Germany) containing endothelial growth factors (C-39215, Promocell, Heidelberg, Germany). Upon thawing, cells were initially seeded in T75 cell culture flasks, and then the cells were cultured at 37 °C in an environment with 95% humidity and 5% CO_2_. When the cells reached 80–90% confluence, they were subjected to a 4 min trypsin incubation at 37 °C and then transferred to 10 cm cell culture plates at a seeding density of 5000 cells/cm^2^. For experimental purposes, endothelial cells at passage 7 were used and seeded in 6-well plates. After a 24 h incubation period, the medium was replaced with a fresh medium containing one of the following: ethanol (400 mM), Rapalink-1 (250 pM), or a combination of 400 mM ethanol and Rapalink-1 (250 pM). The medium was refreshed every other day. Rapalink-1 was procured from Biozol. All experiments were performed with three different primary HUVEC models.

### 2.2. Immunofluorescence Staining

HUVECs were seeded in a 96-well plate (5000 cells/cm^2^). The medium was changed the next day with a new medium alone (for control) or a medium containing 400 mM ethanol, 250 pM Rapalink-1, and a combination of 400 mM ethanol and 250 pM Rapalink-1 for 2 h. After that, the cells were washed thrice with PBS and fixed with 4% paraformaldehyde for 10 min. The cells were washed again with PBS and, for permeabilization, the cells were treated with 0.2% Triton™ X-100 for 10 min. Successively, the cells were incubated with 5% bovine serum albumin (BSA) for 1 h at RT. The cells were incubated with primary antibodies H2A-X (1:500, Cat. 80312S, Cell Signaling, Danvers, MA, USA) and 8-OHDG (1:500, Cat. No. BSS-BS-1278R, BIOSS, Woburn, MA, USA) overnight at 4 °C. The following day, the cells were washed thrice with PBS and then incubated with secondary antibodies (1:1000, Alexa Fluor 488, Cat. No.: 4408 and 1:100, Alexa Fluor 594, Cat. No.: 8889, Cell signaling, Danvers, MA, USA) for 1 h at RT. DAPI (cat no: 62248, Thermo Fisher Scientific, Waltham, MA, USA) was used for nuclear staining. The images were taken at 20× magnification using a Leica DMi8 Inverted Microscope and the compatible LAS-X Life Science Microscope Software (Leica Application Suite X) Platform. ImageJ (version 1.53c) (National Institutes of Health, Bethesda, MD, USA) was used for the analysis of images.

### 2.3. Western Blot

For protein expression analysis, endothelial cells underwent different treatments, including exposure to 400 mM ethanol, Rapalink-1 (250 pM), or a combination of 400 mM ethanol and Rapalink-1 (250 pM). Untreated endothelial cells were used as controls. Following a 24 h treatment period, the total protein content was extracted using a RIPA buffer. The protein concentration was quantified colorimetrically using the DC Protein Assay Kit (500–0116, Bio-Rad, Hercules, CA, USA) in accordance with the manufacturer’s instructions. Measurements were taken with the Paradigm micro-plate reader. Subsequently, 25 µg of total protein under reducing conditions was loaded onto a 12% sodium dodecyl sulfate–polyacrylamide gel. Electrophoresis was conducted at 60 Volts for 20 min, followed by 110 Volts for 30–60 min. The separated proteins were then transferred onto a 0.45 µm pore size nitrocellulose membrane at 250 mA for 120 min. To minimize nonspecific binding, the membranes were blocked for one hour with a 5% bovine serum albumin (BSA) solution in 0.05% TBST. For primary antibody incubation (details provided in Supplementary Table S1), the membranes were placed overnight at 4 °C on a shaking platform, and primary antibodies were diluted in a blocking solution containing 5% BSA. Afterward, the membranes underwent 3 × 10 min washes with TBST and were subsequently exposed to secondary antibodies diluted in 0.05% TBST (refer to Supplementary Table S1) for one hour at room temperature. All antibody dilutions were prepared in Tris-buffered saline with Tween20 (TBST). Densitometry analysis was performed using NIH-ImageJ with β-actin correction.

### 2.4. Quantitative PCR

For quantitative PCR (qPCR) analysis, total RNA extraction was carried out using the Nucleo Spin RNA kit (740955.50, MACHEREY-NAGEL, Düren, Germany) according to the manufacturer’s protocols for two distinct sets of endothelial cells: one being untreated endothelial cells (control), and the other comprising endothelial cells exposed to either 400 mM ethanol or a combination of 400 mM ethanol with Rapalink-1 (250 pM) for 24 h. A total of 1.2 µg of RNA was utilized for reverse transcription, accomplished using the M-MLV Reverse Transcriptase kit (M1701, Promega, Walldorf, Germany), Random Hexamer Primers (48190011, Thermo Fisher), and RiboLock RNase Inhibitor (EO0384, Thermo Fisher). The qPCR assays were conducted employing the AceQ SYBR qPCR Master Mix (Q111-03, Vayzme, Nanjing, China) on a Bio-Rad thermal cycler. The thermal cycling program consisted of an initial denaturation step at 95 °C for 8 min, followed by 40 cycles of 95 °C for 15 s, 58.9 °C for 30 s, and 72 °C for 30 s, concluding with a melting curve analysis. To calculate relative mRNA expression, data were normalized to β-actin expression, and the relative expression levels were quantified using the comparative ΔCT method. Primer sequences are provided in Supplementary Table S2.

### 2.5. β-Galactosidase Staining

Senescence Cells Histochemical Staining Kit (GALS, Sigma, MO, USA) was used for β-Gal staining following the manufacturer’s instructions. Endothelial cells were subjected to treatments including exposure to either 400 mM ethanol or a combination of 400 mM ethanol with Rapalink-1 (250 pM), while the control group remained untreated. After 24 h, the cells were fixed with a subsequent exposure to SA–beta-galactosidase staining solution. This staining procedure took place at 37 °C for seven hours, after which the staining solution was aspirated. The cells were then overlayed with a 70% glycerol solution and stored at 4 °C. Microscopic images of the stained cells were acquired, and the quantification of stained cells was conducted using Image J software (version 1.53c).

### 2.6. Statistical Analysis

We employed a one-way ANOVA followed by Tukey’s post hoc test for the analysis of multiple groups. The significance threshold was set to * *p* < 0.05.

## 3. Results

### 3.1. Rapalink-1 Inhibits Ethanol-Induced Cellular Senescence

There is no single trait that can define cellular senescence. To confirm a senescent phenotype in cells, the presence of three characteristics has been suggested. First, senescent cells stop growing and show cell cycle arrest, which is confirmed by the increased expression of senescence markers such as P21. Second, the senescent cells show structural changes that can be validated through β-gal staining and loss of Lamin B1. Third, senescent cells increase the expression of SASP factors and show an increase in ROS levels. To confirm occurrence of ethanol-induced senescence in endothelial cells, all of these characteristics were validated.

**Figure 1:**
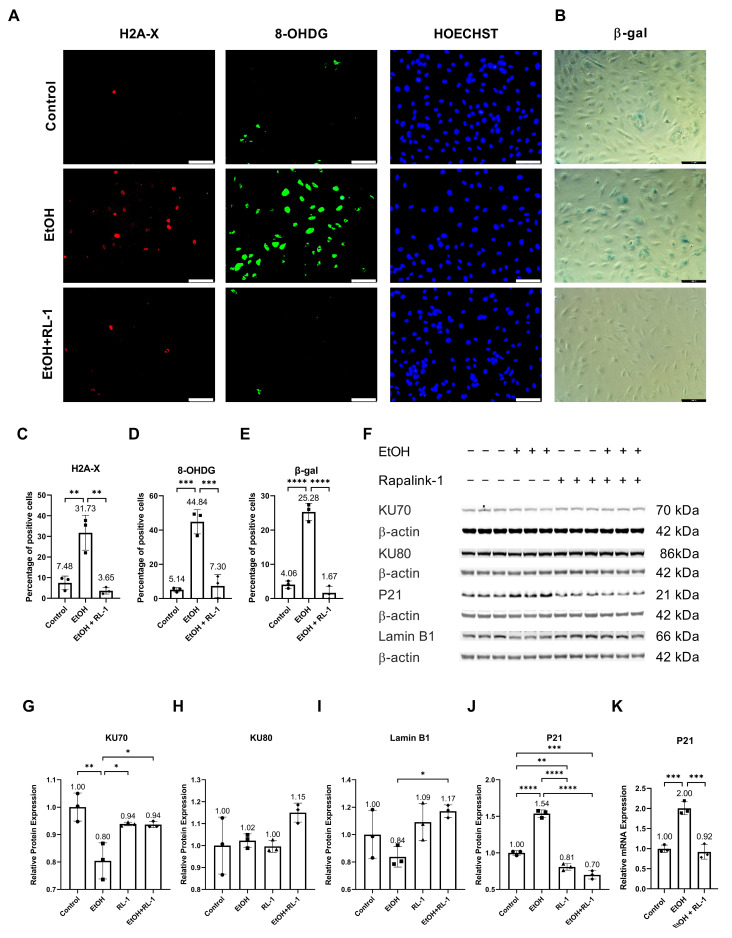
Rapalink-1 inhibited ethanol-induced DNA damage and senescence. Immunofluorescence staining for (**A**) H2A-X and 8-OHDG. (**B**) β-gal staining. Quantification of immunofluorescence for (**C**) H2A, (**D**) 8-OHDG, and (**E**) β-gal staining. (**F**) Western blot showing protein expression of KU70, KU80, P21, and Lamin B1. Relative protein expression of (**G**) KU70, (**H**) KU80, (**I**) Lamin B1, and (**J**) P21. (**K**) Relative mRNA expression of P21. β-actine was used as a loading control. The experiment was performed in triplicates. The data were analyzed using one-way ANOVA followed by Tukey’s test. RL-1 (Rapalink-1) *n* = 3, * *p* < 0.05, ** *p* < 0.01, *** *p* < 0.001, **** *p* < 0.0001, scale bar = 100 µm.

Ethanol metabolism produces oxidative stress [24], which can cause DNA damage [2]. To investigate whether Rapalink-1 can ameliorate oxidative-stress-induced DNA damage in endothelial cells exposed to ethanol, immunofluorescence staining was performed for H2A-X and 8-OHDG. Both stainings showed ethanol-induced DNA damage (Figure 1A,C,D). Rapalink-1 ameliorated immunofluorescence staining for H2A-X, a percentage of positive cells (Control = 7.48 ± 3.28, EtOH = 31.73 ± 8.45, EtOH + Rapalink-1 = 3.65 ± 1.61, *n* = 3, * *p* < 0.01, Figure 1C) and 8-OHDG, a percentage of positive cells (Control = 5.14 ± 1.25, EtOH = 44.84 ± 7.05, EtOH + Rapalink-1 = 7.30 ± 6.62, *n* = 3, ** *p* < 0.001, Figure 1D) in ethanol treated endothelial cells. Rapalink-1 also blocked ethanol-induced senescence in endothelial cells and a percentage of β-gal positive cells (Control = 4.06 ± 1.06, EtOH = 25.28 ± 2.54, EtOH + Rapalink-1 = 1.67 ± 1.88, *n* = 3, *** *p* < 0.0001, Figure 1B,E). Rapalink-1 improved the relative protein expression of DNA repair protein KU70 (Figure 1F,G, Table 1) and aging marker Lamin B1 (Figure 1F,I, Table 1). Neither treatment affected the relative protein expression of DNA repair protein KU80 (Figure 1F,H, Table 1). Rapalink-1 also mitigated the relative protein and mRNA expression of the senescence marker P21 (Figure 1F,J,K, Table 1 and Table 2).

### 3.2. Pathway Analysis

**Figure 2:**
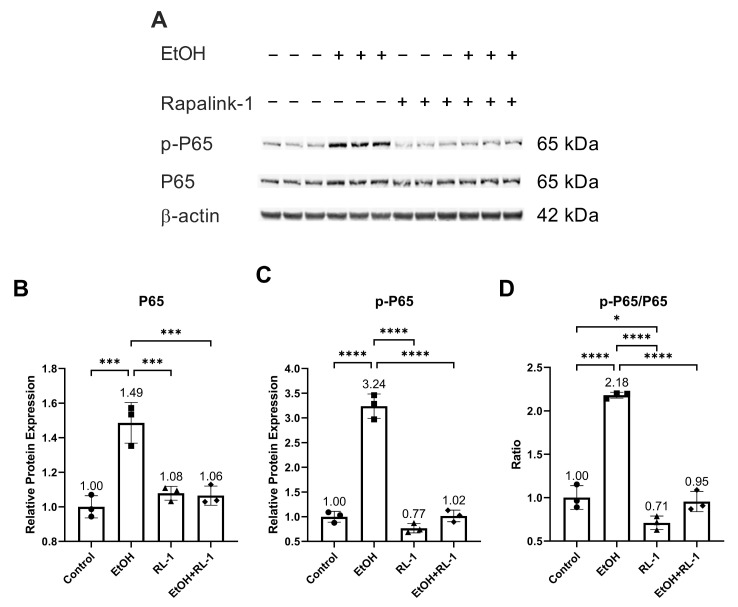
Rapalink-1 inhibited ethanol-induced activation of NF-κB. (**A**) Western blot showing protein expression of NF-κB subunit P65 and phosphorylated P65. Relative protein expression of NF-κB subunit (**B**) P65 and (**C**) p-P65. (**D**) Ratio of p-P65/P65. β-active was used as a loading control. The experiment was performed in triplicates. The data were analyzed using one-way ANOVA followed by Tukey’s test. RL-1 (Rapalink-1), *n* = 3, * *p* < 0.05, *** *p* < 0.001, **** *p* < 0.0001.

To investigate the activation of pathways of interest, including NF-κB, MAPKs, and mTOR pathways, a Western blot was performed for protein analysis. Rapalink-1 inhibited the increase in the protein expression and phosphorylation of NF-κB subunit P65 in endothelial cells treated with ethanol for 24 h (Figure 2A–C and Table 2). Also, Rapalink-1 lowered the ratio of p-65/P65 in untreated and ethanol-treated endothelial cells (Figure 2D and Table 2).

**Figure 3:**
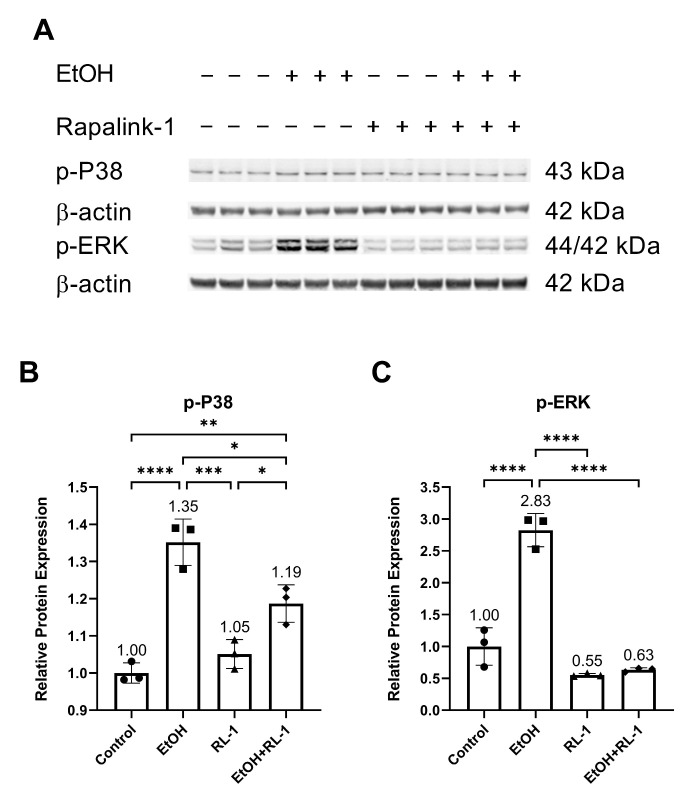
Rapalink-1 inhibited ethanol-induced activation of P38 and ERK. (**A**) Western blot showing protein expression of p-P38 and p-ERK. Relative protein expression of (**B**) p-P38 and (**C**) p-ERK. β-actine was used as a loading control. The experiment was performed in triplicates. The data were analyzed using one-way ANOVA followed by Tukey’s test. RL-1 (Rapalink-1), *n* = 3, * *p* < 0.05, ** *p* < 0.01, *** *p* < 0.001, **** *p* < 0.0001.

Next, we investigated the activation of MAPKs. Previously, ethanol treatment has been shown to increase the activation of P38 and ERK [2]. Confirming the already reported results, ethanol increased the relative protein expression of p-P38 and p-ERK, which was attenuated by the Rapalink-1 treatment (Figure 3A–C and Table 2).

**Figure 4:**
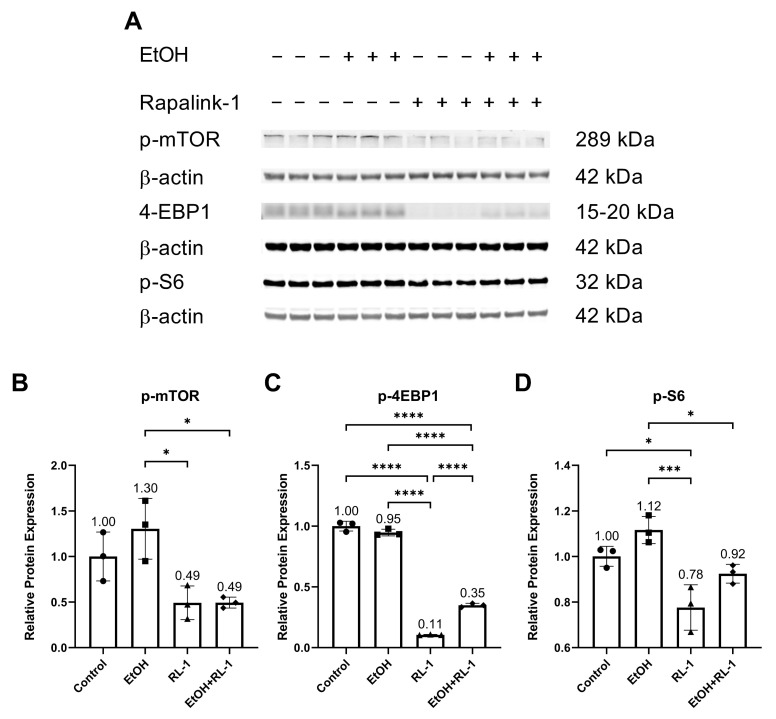
Rapalink-1 inhibits the activation of the mTOR pathway. (**A**) Western blot showing protein expression of p-mTOR, p-4-EBP1, and p-S6. Relative protein expression of (**B**) p-mTOR, (**C**) p-4EBP1, and (**D**) p-S6. β-actine was used as a loading control. The experiment was performed in triplicates. The data were analyzed using one-way ANOVA followed by Tukey’s test. RL-1 (Rapalink-1), *n* = 3, * *p* < 0.05, *** *p* < 0.001, **** *p* < 0.0001.

The mTOR pathway is known to play an important role in aging and cellular senescence. Protein analysis showed that ethanol alone did not significantly increase the relative protein expression of p-mTOR and p-S6 (Figure 4A,B,D, and Table 2). Rapalink-1 alone and Rapalink-1 combined with ethanol significantly reduced the relative protein expression of p-mTOR, p-4EBP1, and p-S6 compared to endothelial cells treated only with ethanol (Figure 4B–D, and Table 2). Rapalink-1 alone also significantly reduced the relative protein expression of p-4EBP1 and p-S6 relative to the untreated controls (Figure 4C,D and Table 2).

### 3.3. Rapalink-1 Mitigated the Expression of SASP Factors

**Figure 5:**
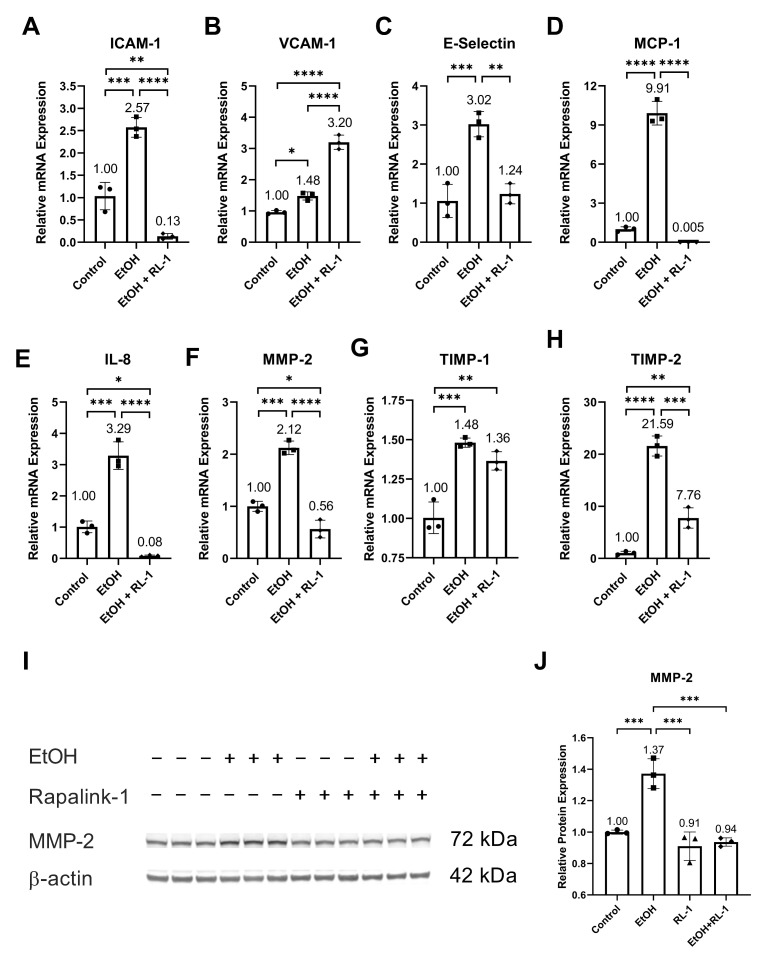
Rapalink-1 mitigated mRNA expression of SASP factors and MMP2. Relative mRNA expression of (**A**) ICAM-1, (**B**) VCAM-1, (**C**) E-Selectin, (**D**) MCP-1, (**E**) IL-8, (**F**) MMP-2, (**G**) TIMP-1, and (**H**) TIMP-2. (**I**) Western blot showing protein expression of MMP-2. (**J**) Relative protein expression of MMP-2. β-actine was used as a loading control. The experiment was performed in triplicates. The data were analyzed using one-way ANOVA followed by Tukey’s test. RL-1 (Rapalink-1), *n* = 3, * *p* < 0.05, ** *p* < 0.01, *** *p* < 0.001, **** *p* < 0.0001.

Ethanol increased the relative mRNA expression of SASP factors, including ICAM-1, VCAM-1, E-Selectin, MCP-1, IL-8, MMP-2, and TIMP-2 (Figure 5A–F,H, and Table 2). It also increased the relative protein expression of MMP-2 (Figure 5I,J and Table 1). Neither treatment affected the relative mRNA expression of TIMP-1. Rapalink-1 ameliorated the ethanol-induced relative mRNA expression of ICAM-1, E-Selectin, MCP-1, IL-8, MMP-2, and TIMP-2 (Figure 5A,C–F,H and Table 2). It also attenuated the relative protein expression of MMP-2 (Figure 5I,J, Table 1).

## 4. Discussion

Cardiovascular risk factors, including smoking, alcohol abuse, hypertension, and oxidative stress, cause cellular senescence and induce SASP [1,2,3]. Cellular senescence and SASP are known to contribute to cardiovascular diseases. In this study, we used Rapalink-1 to mitigate ethanol-induced endothelial cell senescence and SASP. Moreover, we investigated the impact of Rapalink-1 on the activation of NF-κB, MAPKs, and mTOR pathways in ethanol-treated endothelial cells.

The ethanol metabolism produces oxidative stress [24], which can cause oxidative-stress-induced DNA damage [2]. Confirming the previously reported findings, ethanol treatment resulted in oxidative-stress-induced DNA damage (Figure 1A,C,D) [2], which was diminished by Rapalink-1 treatment. As ethanol treatment and oxidative-stress-induced DNA damage can result in cellular senescence [1,2,3], β-gal staining was performed to visualize senescence in endothelial cells treated with ethanol. Increased β-gal activity is one of the characteristics of senescent cells [4,5]. The increased β-gal activity was observed in ethanol-treated endothelial cells (Figure 1B,E). The Rapalink-1 treatment reduced β-gal activity in endothelial cells exposed to ethanol (Figure 1B,E) and improved the relative protein expression of DNA repair protein KU70 (Figure 1G) and aging marker Lamin B1 (Figure 1I). KU70 forms a heterodimer with KU80 and repairs double-stranded DNA breaks [25]. Lamin B1 maintains nuclear stability and regulates DNA replication and gene transcription. Lamin B1 decreases in senescent cells [26,27], and the silencing of Lamin B1 can induce premature senescence [26]. The reduced stability of Lamin B1 mRNA causes Lamin B1 loss in senescence [27]. Senescent cells exhibit cell cycle arrest, which was confirmed by the increased expression of P21 in endothelial cells treated with ethanol (Figure 1J,K). P21 is a cyclin-dependent kinase (CDK) inhibitor, a negative regulator of cell cycle progression [7]. Its induction results in indefinite growth arrest and establishes a senescence state [7,12]. Rapalink-1 also mitigated the relative protein and mRNA expression of P21 (Figure 1J,K, Table 1 and Table 2). These findings suggest that Rapalink-1 inhibited senescence in ethanol-treated endothelial cells by suppressing oxidative stress-induced DNA damage, improving the expression of KU70 and Lamin B1, and attenuating P21 expression.

The pathway analysis showed that Rapalink-1 inhibited the activation of NF-κB (Figure 2), MAPKs (Figure 3), and mTOR (Figure 4) pathways in endothelial cells exposed to ethanol. These pathways are activated in senescent cells [1,2,3] and are known to contribute to cellular aging and senescence [11,14]. Oxidative stress and DNA damage can also activate P38 and ERK, consequently triggering cellular senescence [14]. NF-κB, P38, and ERK increase the expression of P21 by increasing its transcription [14,28]. P38 also elevates P21 levels by increasing P21 mRNA stability [14]. Inhibiting senescent signals by blocking P38 can improve Lamin B1 expression [27]. Moreover, mTOR downstream signaling molecule p-4EBP1 stabilizes the P21 protein [29]. Taken together, these findings suggest that Rapalink-1, by inhibiting the activation of NF-kB, P38, ERK, and 4EBP1, alleviated Lamin B1 expression and suppressed the expression of P21. Previous studies have shown that blocking the activation of NF-κB, MAPKs, and mTOR can dampen senescence in various cell types in vivo and in vitro [17,18,19]. The accumulation of senescent cells has been observed in atherosclerotic lesions [6,7,13]. By eliminating senescent cells, atherosclerosis progression, atherosclerotic plaque rupture, fibrosis, aortic calcification, and cardiomyocyte hypertrophy could be reduced [6,13]. Moreover, clearance of senescent cells could promote cardiomyocyte regeneration, improve systolic cardiac function and vascular relaxation, and extend the health span [6]. Previously, mTOR has been shown to be the upstream regulator of NF-κB, and mTOR inhibition by rapamycin and shmTOR has been found to suppress NF-κB activation in mouse HT-22 hippocampal neuronal cells [30] and mTOR siRNA inhibited NF-κB activation in macrophages [31], treated with high glucose. Similar to rapamycin, an inhibitor of mTOR, blocking NF-κB activation [30], in our study, Rapalink-1, an mTOR inhibitor, inhibited NF-kB activation in endothelial cells exposed to H_2_O_2_ (Figure 2). Moreover, mTOR can increase P38 activation and, via P38 activation, can upregulate senescence markers including P53 and P16 [32]. Taken toghether, this suggests that Rapalink-1, by inhibiting the activation of NF-κB (Figure 2), MAPKs (Figure 3), and mTOR (Figure 4), can suppress senescence (Figure 1) and can thus be beneficial against cardiovascular diseases [6].

Furthermore, in clinical and experimental animal studies, increased activation of NF-κB, MAPKs, and mTOR has been observed in IA and atherosclerotic tissue [20,21,23,33]. NF-κB subunit P50 deficiency in mice, NF-κB decoy in rats, and the blocking of nuclear import of NF-κB in mice has been found to impair IA formation and reduce atherosclerotic lesion size [20,21,23]. Additionally, blocking the nuclear import of NF-κB was found to increase anti-inflammatory M2 macrophages in atherosclerotic lesions [21]. In human IA tissue, MAPK phosphorylation is co-related with IA size [33], suggesting its role in IA growth and rupture. In animal experimental studies, mTOR inhibitor and mTOR-siRNA reduced atherosclerotic plaque vulnerability and rupture [15,22]. Therefore, it can be postulated that Raplink-1, by inhibiting the activation of NF-κB, MAPKs, and mTOR pathways (Figure 2, Figure 3 and Figure 4), can be a potential drug candidate against atherosclerosis and IAs. In addition to that, in experimental animal studies, mTOR inhibition, NF-kB deficiency, NF-κB decoy, and the blocking of nuclear import of NF-κB has been found to reduce macrophage infiltration and the expression of ICAM-1, MCP-1, IL-8, and MMP-2 [15,20,21]. The increased expression of SASP factors is one of the characteristics of senescent cells. The increased mRNA expression of SASP factors was validated in endothelial cells exposed to ethanol (Figure 5). The mRNA expression of these SASP factors was mitigated by Rapalink-1 in ethanol-treated endothelial cells (Figure 5). The expression of SASP factors in senescent cells is upregulated by NF-κB transcription activity [11], where MAPKs are upstream regulators of NF-kB [10,14]. P38 activation is required for SASP expression and its inhibition can mitigate SASP expression [34]. Moreover, inhibition of P38 activation can reduce NF-κB transcription activity [34], resulting in reduced expression of SASP factors. This suggests that Rapalink-1 ameliorated the expression of SASP factors by inhibiting the activation of NF-kB, P38, and ERK in ethanol-treated endothelial cells.

The expression and release of SASP factors from senescent cells contribute to cardiovascular diseases through various mechanisms. The release of MCP-1 and IL-8 facilitates the recruitment of inflammatory cells, where cell adhesion molecules like ICAM-1 and E-selectin support the adhesion of inflammatory cells to the vascular wall, and MMP-2 release can promote their infiltration [10]. The infiltrated inflammatory cells release pro-inflammatory molecules and MMPs, resulting in the exacerbation of inflammation and degeneration of vascular tissue [10]. Rapalink-1, by inhibiting the expression and release of cell adhesion molecules, chemokines, and MMPs (Figure 5), can attenuate the infiltration and recruitment of inflammatory cells. MCP-1 deficiency in mice and the blocking of MCP-1 in rats have been shown to decrease the size and progression of atherosclerotic lesions, impair IAs formation and progression, inhibit macrophage infiltration, and mitigate the expression of MMP2 [35,36]. MMPs degenerate extracellular matrix. Increased MMP-2 expression has been observed in IA walls and atherosclerotic lesions [37,38]. Inhibiting MMP2 and MMP-9 was found to reduce the progression of IAs [37], and MMP-2 deficiency was found to reduce atherosclerotic lesion formation [39]. Furthermore, MMPs can modulate inflammatory response through their proteolysis activity on cell adhesion molecules, chemokines, and cytokines [40]. These findings suggest that Rapalink-1, by mitigating the expression of SASP factors, can dampen inflammation and tissue modulation, suggesting its therapeutic potential in cardiovascular diseases.

## 5. Conclusions

Senescence in ethanol-treated endothelial cells was confirmed by validating structural changes through increased β-gal activity and Lamin B1 loss, cell cycle arrest via increased expression of P21, and increased expression of SASP factors as secondary markers. All these characteristics demonstrated ethanol-induced senescence in endothelial cells. Rapalink-1 mitigated ethanol-induced senescence in endothelial cells. It reduced ethanol-caused oxidative-stress-induced DNA damage and inhibited the activation of NF-κB, MAPKS, P38, and ERK.

## Figures and Tables

**Table 1:** Quantification of relative protein expression. The data were analyzed using one-way ANOVA followed by Tukey’s test.

Protein	Treatment Duration	Control	EtOH	Rapalink-1	EtOH + Rapalink-1	*p*-Value
KU70	24 h	1.00 ± 0.05	0.80 ± 0.06	0.94 ± 0.01	0.94 ± 0.01	0.0028
KU80	24 h	1.00 ± 0.03	1.02 ± 0.03	1.00 ± 0.03	1.15 ± 0.04	0.0874
Lamin B1	24 h	1.00 ± 0.18	0.84 ± 0.07	1.09 ± 0.14	1.17 ± 0.04	0.0433
P21	24 h	1.00 ± 0.03	1.54 ± 0.05	0.81 ± 0.04	0.70 ± 0.06	0.0001
P65	24 h	1.00 ± 0.06	1.49 ± 0.12	1.08 ± 0.04	1.06 ± 0.06	0.0002
p-P65	24 h	1.00 ± 0.11	3.24 ± 0.25	0.77 ± 0.09	1.02 ± 0.12	0.0001
p-P65/P65	24 h	1.00 ± 0.14	2.18 ± 0.03	0.71 ± 0.08	0.95 ± 0.12	0.0001
p-p38	24 h	1.00 ± 0.03	1.35 ± 0.06	1.05 ± 0.04	1.19 ± 0.05	0.0001
p-ERK	24 h	1.00 ± 0.29	2.83 ± 0.26	0.55 ± 0.02	0.63 ± 0.03	0.0001
p-mTOR	24 h	1.00 ± 0.27	1.30 ± 0.33	0.49 ± 0.18	0.49 ± 0.06	0.0067
p-S6	24 h	1.00 ± 0.04	1.12 ± 0.06	0.78 ± 0.10	0.92 ± 0.04	0.0014
p-4EBP1	24 h	1.00 ± 0.04	0.95 ± 0.03	0.11 ± 0.003	0.35 ± 0.02	0.0001
MMP-2	24 h	1.00 ± 0.01	1.37 ± 0.09	0.91 ± 0.09	0.94 ± 0.03	0.0001

**Table 2:** Quantification of Relative mRNA expression. The data were analyzed using one-way ANOVA followed by Tukey’s test.

Gene	Control	EtOH	EtOH + Rapalink-1	*p*-Value
P21	1.00 ± 0.09	2.00 ± 0.16	0.92 ± 0.18	0.0002
IL-8	1.00 ± 0.19	3.29 ± 0.44	0.08 ± 0.03	0.0001
MCP-1	1.00 ± 0.18	9.91 ± 0.91	0..005 ± 0.004	0.0001
ICAM-1	1.00 ± 0.30	2.57 ± 0.22	0.13 ± 0.06	0.0001
VCAM-1	1.00 ± 0.06	1.48 ± 0.13	3.20 ± 0.23	0.0001
E-Selectin	1.00 ± 0.42	3.02 ± 0.32	1.24 ± 0.26	0.0007
MMP-2	1.00 ± 0.10	2.12 ± 0.13	0.56 ± 0.17	0.0001
TIMP-1	1.00 ± 0.10	1.48 ± 0.03	1.36 ± 0.06	0.0004
TIMP-2	1.00 ± 0.32	21.59 ± 1.92	7.76 ± 1.96	0.0001

## Data Availability

The data is contained within the article.

## Supplementary Materials

The following supporting information can be downloaded at: https://www.mdpi.com/article/10.3390/cells12222609/s1, Table S1: Primary and Secondary antibodies; Table S2: Primer list.
