# Supplementary material for: mTOR Inhibitor Rapalink-1 Prevents Ethanol-Induced Senescence in Endothelial Cells
Source: Cells. 2023 Nov 11;12(22):2609. doi: 10.3390/cells12222609 (PMC10670449; doi:10.3390/cells12222609)
Supplement: Supplementary file 1 [file cells-12-02609-s001.zip › cells-2645178-supplementary.pdf]

Table S1. Primary and Secondary antibodies.

Antibody	MW(kDa)	Brand	Catalog Number	Concentration
P21	21	Cell Signaling	# 2947S	1:1,000
Lamin B1	66	Abcam	ab16048	0.1 µg/mL
KU80	86	Cell Signaling	#2753S	1:1,000
Ku70	70	Cell Signaling	#4588S	1:1,000
P65	65	Cell Signaling	#6956S	1:1,000
p-P65	65	Cell Signaling	#3033S	1:1,000
MMP-2	70	Invitrogen	436000	2 µg/mL
β-actin (Rabbit)	45	Cell Signaling	#4970S	1:1,000
β-actin (Mouse)	45	Invitrogen	#MA5-15739	1:1,000
p-P38	43	Cell Signaling	#4511S	1:1,000
p-JNK	46,54	Cell Signaling	#9255S	1:2,000
p-ERK	42,44	Cell Signaling	#4370S	1:2,000
p-mTOR	289	Cell Signaling	#2971S	1:1,000
p-S6	32	Cell Signaling	#2215S	1:1,000
p-4EBP1	15-20	Cell Signaling	#2855S	1:1,000
8-OHDG		BIOSS	BSS-BS-1278R	1:500
Alexa Fluor Plus 488		Cell signaling	4408	1:1,000
Alexa Fluor 594		Cell signaling	8889	1:1,000
IRDye 800CW Goat-anti-Rabbit Antibody		LiCor	#926-32211	1:10,000
IRDye 680RD Donkey anti-Mouse IgG Secondary Antibody		LiCor	#926-68072	1:10,000

Table S2. Primer list.

Target gene	Gene Accession Number	Sense 5' -3'	Antisense 5' -3'
ICAM-1	NM_000201	CACAGTCACCTATGGCAACGA	TGGCTTCGTCAGAATCACGTT
VCAM-1	NM_080682, NM_001199834, NM_001078	AGTGGTGGCCTCCTGAATGG	CTGTGTCTCCTGTCTCCGCT
IL-8	NM_001354840, NM_000584	TGCCAAGGAGTGCTAAAG	CTCCACAACCCTCTGCAC
MCP-1	NM_002982	CACCAATAGGAAGATCTCAGTGC	TGAGTGTTCAAGTCTTCGGAGTT
MMP2	NM_001302510, NM_001302509, NM_001127891, NM_004530, NM_001302508	ATAACCTGGATGCCGTCGT	AGGCACCCTTGAAGAAGTAGC
TIMP1	NM_003254	TGGCTTCTGGCATCCTGTTGTTG	CGCTGGTATAAGGTGGTCTGGTTG
TIMP2	NM_003255	GAATCGGTGAGGTCCTGTCCTGA	CCTGCACACAAGCCCGGATAAA
E-selectin	NM_000450	CAAGAAGAAGCTTGCCCTATG	ACTTGAGTCCACTGAAGCCA
P21	NM_001374511, NM_001220777, NM_001374510, NM_001374512, NM_001374513, NM_001291549, NM_078467, NM_001374509, NM_001220778, NM_000389	GACACCACTGGAGGGTGACT	CAGGTCCACATGGTCTTCCT
